# Establishment Failure in Biological Invasions: A Case History of *Littorina littorea* in California, USA

**DOI:** 10.1371/journal.pone.0016035

**Published:** 2011-01-10

**Authors:** Andrew L. Chang, April M. H. Blakeslee, A. Whitman Miller, Gregory M. Ruiz

**Affiliations:** Smithsonian Environmental Research Center, Edgewater, Maryland, United States of America; University of Canterbury, New Zealand

## Abstract

**Background:**

The early stages of biological invasions are rarely observed, but can provide significant insight into the invasion process as well as the influence vectors have on invasion success or failure.

**Methodology/Principal Findings:**

We characterized three newly discovered populations of an introduced gastropod, *Littorina littorea* (Linné, 1758), in California, USA, comparing them to potential source populations in native Europe and the North American East Coast, where the snail is also introduced. Demographic surveys were used to assess spatial distribution and sizes of the snail in San Francisco and Anaheim Bays, California. Mitochondrial DNA was sequenced and compared among these nascent populations, and various populations from the North American East Coast and Europe, to characterize the California populations and ascertain their likely source. Demographic and genetic data were considered together to deduce likely vectors for the California populations. We found that the three large California *L. littorea* populations contained only adult snails and had unexpectedly high genetic diversity rather than showing an extreme bottleneck as typically expected in recent introductions. Haplotype diversity in Californian populations was significantly reduced compared to European populations, but not compared to East Coast populations. Genetic analyses clearly suggested the East Coast as the source region for the California introductions.

**Conclusions and Significance:**

The California *L. littorea* populations were at an early, non-established phase of invasion with no evidence of recruitment. The live seafood trade is the most likely invasion vector for these populations, as it preferentially transports large numbers of adult *L. littorea*, matching the demographic structure of the introduced California *L. littorea* populations. Our results highlight continued operation of live seafood trade vectors and the influence of vectors on the demographic and genetic structure of the resulting populations, especially early stages of the invasion process.

## Introduction

The prediction and prevention of biological invasions is an evolving field, but significant gaps still hinder our understanding of events during the early stages of invasions, a crucial point where many would-be invasions fail [1]–[3]. Early invasion stages are rarely documented because most invasions are discovered only when they have become abundant enough to be conspicuous to the casual observer [4], [5]. Such introductions may go unnoticed for years to decades, and often little or no historical information exists regarding their initial arrival, establishment and spread [4], [6]. In addition, many newly introduced populations simply fail to establish self-sustaining populations and are never documented [7], [8]. Consequently, we lack robust information on the early stages of most introductions, whether successful or not, even though they may provide essential information on the vectors transporting the species as well as the invasion process itself.

In marine systems, past studies have often compared the genetic structure of introduced to native populations at various time horizons after establishment. These analyses have been used to infer potential source populations, invasion histories, and vectors of introduction [9]–[11]; see review in Geller et al. (2010) [12]. In addition, there has been considerable attention to shifts in genetic structure from native (source) to introduced (recipient) populations. Recently introduced, self-sustaining populations of non-native species are often observed to have significantly lower genetic diversity compared to their source populations, although recent evidence indicates that such bottlenecks may not be as common as would be theoretically expected, especially since introductions are not always single occurrence events but may be the result of multiple inoculations [10].

We describe demographic and genetic characteristics for recently detected populations of the marine snail *Littorina littorea* (Linné, 1758) at multiple locations in California, USA. As outlined here, these populations are at a very early stage of the invasion process and do not yet appear to be established. Thus, this is a useful model system for characterizing early-stage invasions; specifically, our study examined possible source location(s), timing of introduction(s), putative vector(s), and genetic structure across native and introduced populations. Finally, we assessed the likelihood of *L. littorea* eventually establishing self-sustaining populations along the North American West Coast.

## Materials and Methods

### *Study system*: Littorina littorea

The common European Atlantic periwinkle *Littorina littorea* is an omnivorous, grazing intertidal gastropod that was accidentally introduced to the North American East Coast within the last few centuries [11], [13]–[15], where it is now extraordinarily abundant on New England rocky shores [15], [16]. *L. littorea* has been shown to fundamentally change North Atlantic intertidal ecosystems via grazing activities, altering the distribution and abundance of algae on rocky shores and converting soft-sediment habitats to hard substrates [17], [18], as well as competitively displacing some native species [19], [20]. *L. littorea* is oviparous, reproducing annually with internal fertilization of egg capsules that are then shed directly into the sea, leading to a planktotrophic larval development time of four to seven weeks [13], [21], [22].

On the West Coast of North America, singletons or small numbers of *L. littorea* have occasionally been reported over the past seventy years, mostly from California, but no established populations have ever been recorded [23], [24] (Table 1). We report here on three larger populations recently discovered in San Francisco Bay (2002, 2007) and Anaheim Bay (2002).

**Table 1:** *Littorina littorea* found on the Pacific Coast of North America.

Year	Location	# Live (# Dead)	Collector(s)	Source
1937	Deception Pass, Puget Sound	12 (4)	Chace	Hanna 1966, Carlton 1979
1942	Trinidad Bay		Niles	Carlton 1969
1949	Deception Pass, Puget Sound	6 (2)	Chace	Hanna 1966, Carlton 1979
1966	Newport Beach jetty	1	Smith	Carlton 1979
1968	Berkeley, SF Bay	1	Carlton	Carlton 1969
1968	Southeast Alameda Island, SF Bay	5	Yancey, Kassa	Carlton 1979
1969	South Alameda Island, SF Bay	6	Yancey	Carlton 1979
1969	Bay Farm Island Bridge, SF Bay	1	Yancey	Carlton 1979
1970	Bay Farm Island Bridge, SF Bay	1	Carlton	Carlton 1979
1975	Newport Bay		Carlton	Carlton 1975
1976	Selby, SF Bay	1	Pitt	Carlton 1979
1977	Selby, SF Bay	5	Pitt	Carlton 1979
1995	Hunter's Point, SF Bay	1	Cohen	J. Carlton pers. comm.
1996	Cabrillo Beach breakwater, LA County	2	Yoshimoto	D. Yoshimoto pers. comm.
2001	Coast Guard Island, SF Bay	1	Chang	
2002	Dumbarton Pier, SF Bay	412 (7)	Chang, Ruiz, Drinker, Schofield, Hillmann, Hackman	
2002	Seal Beach, Anaheim Bay	2289 (67)	Chang, Ruiz, Liff-Grieff, Hoang, Pankratz, Kumar, Young	
2003	Berkeley, SF Bay	1	Sousa	E. Grosholz pers. comm.
2004	Oakland, SF Bay	1	Fofonoff	P. Fofonoff pers. comm.
2007	Ashby Spit, SF Bay	140 (4)	Chang, Ruiz, Cohen, Zabin, Attoe	

Year of initial discovery is noted for large populations.

### Demographic surveys and sampling

Intertidal surveys were conducted at 130 locations throughout San Francisco Bay in 2001–2003, 2004–2005, and 2008 to assess the abundance of *Littorina saxatilis*, a smaller Atlantic congener of *L. littorea* that has successfully invaded San Francisco Bay. As *L. saxatilis* individuals inhabit generally similar habitat, have broadly similar morphology, and span the same range of sizes as *L. littorea*, albeit with a smaller maximum adult size, this survey was likely to detect *L. littorea* of any size at a given site. At each site, a presence-absence survey was conducted using teams of 1 to 3 people surveying an area for at least 30 minutes. Sites were chosen according to several criteria, including presence of habitat types known to support *Littorina* spp. [13] and proximity to fishing piers and other sources of discarded algal packing material (the putative vector for *L. saxatilis* ' West Coast introduction [25]), such as seafood restaurants. Surveys were generally conducted at tides below +1 ft mean lower low water, systematically covering the entire intertidal zone between mean high water and the water line and typically covering an area measuring 30 to 50 meters alongshore. Any *L. littorea* encountered during surveys were removed.

Significant *Littorina littorea* populations were found at two locations in San Francisco Bay, at Dumbarton Pier in September 2002 and Ashby Spit in July 2007. The Ashby Spit population was the only one found during a separate project focused on the oyster *Ostrea lurida* at many intertidal sites around the Bay (C. Zabin, pers. comm.).

A large *Littorina littorea* population was reported at Seal Beach in Anaheim Bay (Southern California) in October 2002 (P. Liff-Grieff, pers. comm.). In June 2004, we examined the population and initiated a survey of Anaheim Bay and surrounding areas. Because much of Anaheim Bay is occupied by the U.S. Naval Weapons Station – Seal Beach, access was restricted in many areas, mostly marshy backwater regions, where *L. littorea* may be less likely to occur anyway. Surveys were conducted as extensively as was allowed, using the same protocol as in San Francisco Bay. Additional sites with riprap habitat, mostly jetties and breakwaters, between Los Angeles Harbor and Newport Bay were also surveyed for the presence of *L. littorea* in 2004.

For each of these three large *Littorina littorea* populations, a subset of all *L. littorea* collected were measured (shell height), and another randomly chosen subset was preserved in 95% ethanol for genetic analyses. Ten snails were chosen at random from each population and dissected to assess reproductive status. The remaining snails were fixed in 10% buffered formalin.

Sustained removal efforts were undertaken at each location where large *L. littorea* populations were found and will be reported in a separate manuscript (Chang et al., in prep.).

### DNA sequencing

*Littorina littorea* sequences were from snails collected between 2002 and 2007 in three regions: native Europe, and introduced populations from the North American East Coast, and the North American West Coast (henceforth East Coast and West Coast). European and East Coast sequences were acquired from two studies, Blakeslee et al. (2008) [15] and Brawley et al. (2009) [11], which included 30 European sites (n = 299), ranging from Norway to Spain, and 29 East Coast sites (n = 245), ranging from Labrador to New Jersey (Appendices S1, S2). West Coast samples (n = 102) were collected according to the protocols described above from the three populations reported here in Anaheim Bay (n = 54) and San Francisco Bay (n = 48). Because many of the Ashby Spit specimens were heavily degraded and yielded sequences from just 5 individuals, they were combined with the Dumbarton sequences as a San Francisco Bay grouping in most analyses.

*Littorina littorea* were dissected and the foot tissue removed for DNA analyses. DNA was extracted using a standard CTAB protocol [26]. A 624 base-pair region of the cytochrome b (cyt b) mitochondrial gene was amplified using primers and protocols from Blakeslee et al. (2008) [15]. All sequences were run in both forward and reverse directions and aligned by eye to ensure haplotype identities were accurately assigned. Sequences were aligned using DNAStar (Lasergene, Madison, WI) and Sequencher 4.8 (Gene Codes Corporation, Ann Arbor, MI).

### Molecular Statistical Analyses

Phylogenetic relationships were analyzed using PAUP 4.0 [27], and haplotype identities and frequencies were determined using TCS 1.21 [28]. The haplotype network produced by TCS 1.21 was also used to develop a map showing the relative frequency of haplotypes in North American populations, where they were found, and the connections among haplotypes. Arlequin 3.0 [29] was used to analyze differentiation at the regional, site, and population levels (AMOVA test) and to explore pairwise differences in site-level haplotype frequencies (i.e., pairwise F_ST_). We visualized pairwise F_ST_ results using a multidimensional scaling (MDS) ordination to look for spatial patterns in genetic composition among populations in all three regions. We also used ANOSIM tests in Primer 6 [30] to analyze similarity patterns among regions. Both the MDS ordination and ANOSIM tests were also used to help determine likely source populations/subregions for the West Coast introductions.

Because observed haplotype diversity is affected by sampling effort, we used rarefaction techniques to estimate the true haplotype diversity in each region [15]. Accumulation and estimator curves were calculated using ESTIMATES 8.0 [31]. ESTIMATES uses Monte Carlo re-sampling (through randomization of sample order over many replicates; n = 500 here) to determine the mean accumulation of haplotypes (Sobs) as samples are added over the full data set. We used the Chao2 estimator to predict the eventual asymptote of haplotype diversity for each region [15]. We also used Monte Carlo re-sampling (in ESTIMATES) to standardize samples at the site level at the lowest common individual sampling value (n = 10) to make unbiased comparisons across sites and regions. In several instances, we pooled data from nearby sites to achieve a sampling effort of at least 10 individuals. We used paired Student's t-tests to compare haplotype diversity between regions.

As in-depth comparisons between Europe and the East Coast have been performed in prior studies [11], [14], [15], [32]–[34], our analyses focused on comparisons between the West Coast and the other two regions with some analysis of Europe and the East Coast for comparative purposes only.

## Results

### Demographic surveys

**Figure 1:**
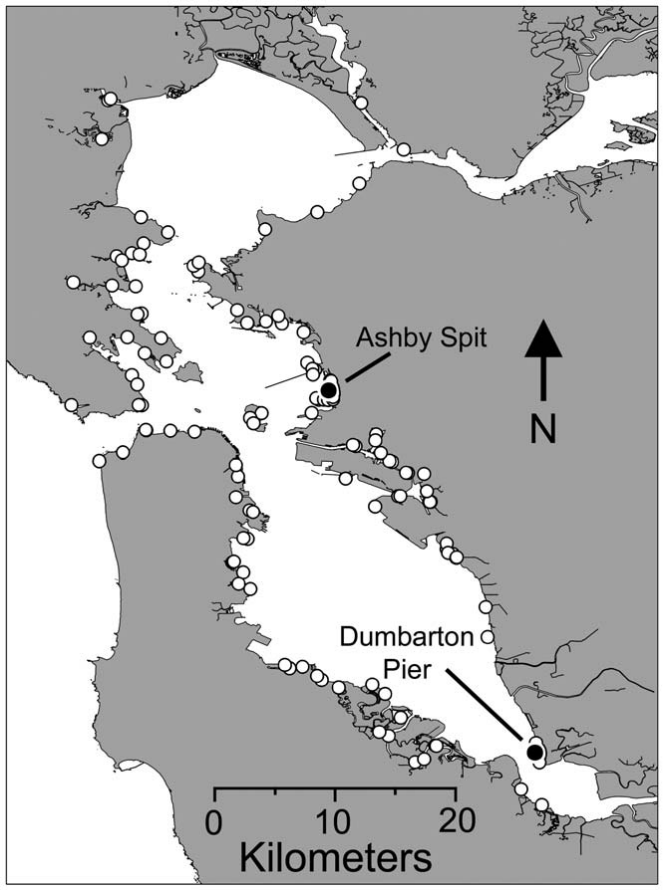
Locations of intertidal surveys in San Francisco Bay (California, USA) completed between 2001 and 2008. Black circles indicate locations of large populations of *Littorina littorea*; white circles indicate locations where *L. littorea* was not detected.

Our intertidal surveys in San Francisco Bay found isolated *L. littorea* individuals along the Oakland shoreline as well as the larger population at Dumbarton Pier. The Ashby Spit population was discovered during work on an unrelated project in 2007 (C. Zabin, pers. comm.), but was not detected in our earlier surveys of that site (Fig. 1).

**Figure 2:**
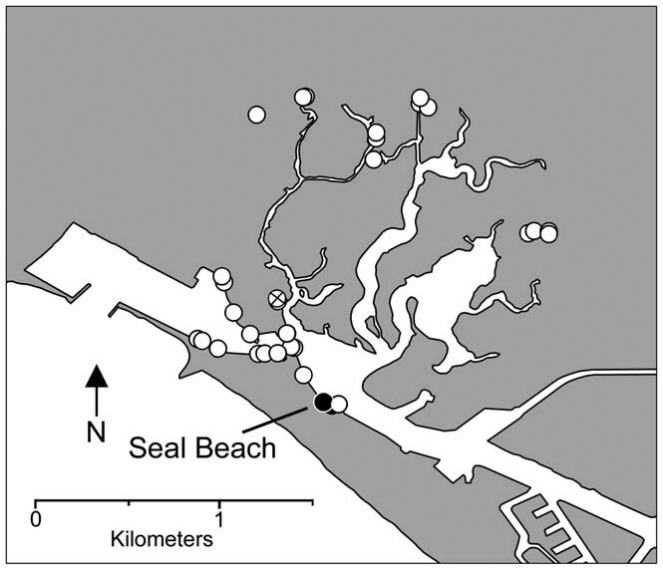
Locations of intertidal surveys in Anaheim Bay (California, USA) completed between 06 June 2004 and 9 December 2004. Black circles indicate locations where *Littorina littorea* was found; white circle with an X indicates the location where only shells (no live individuals) of *L. littorea* were found; open white circles are locations where *L. littorea* was not detected.

No live individuals were found in or near Anaheim Bay aside from the large population at Seal Beach. Military restrictions precluded an exhaustive search of the Bay, but nearly all inaccessible areas were in remote backwater regions far from places where live *L. littorea* and shells were found (Fig. 2).

**Figure 3:**
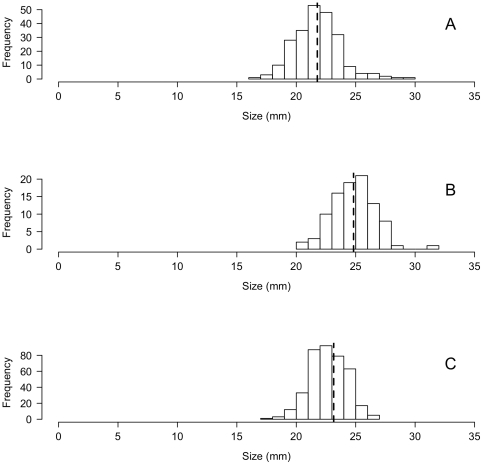
Size-frequency distributions of recently discovered *Littorina littorea* populations. No juveniles are present in any of the three populations. (a) Anaheim Bay (n = 231, mean = 21.77 mm, SD = 1.93 mm), (b) Ashby Spit (n = 94, mean = 24.82 mm, SD = 1.86 mm), and (c) Dumbarton Pier (n = 392, mean = 23.15 mm, SD = 1.58 mm). Not all snails were available for measurement. Dashed line indicates mean.

Although thousands of snails were collected in our extensive surveys, no evidence of successful recruitment was found. All *L. littorea* collected were adults of roughly 20 to 25 mm shell height (Fig. 3), and all dissected individuals from these populations were found to be reproductively mature (i.e. clear evidence of mature gonads and reproductive structures). No newly settled young-of-the-year individuals were ever collected despite the recent presence of large adult populations in both bays. The smallest individual found was at Seal Beach and measured 16 mm in shell height, representing a post-reproductive adult snail.

### Molecular Analyses

#### Spatial distribution of haplotype diversity and source of West Coast populations

**Figure 4:**
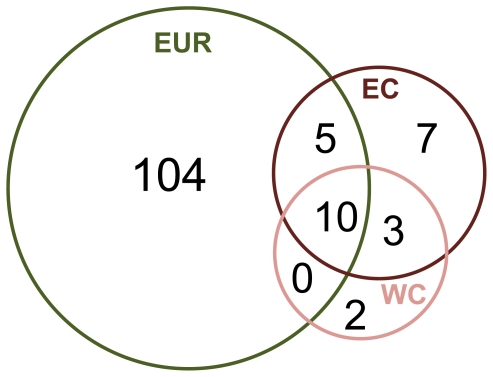
Venn diagram depicting numbers of unique and shared *L. littorea* haplotypes among regions. Regions are Europe (EUR), North American East Coast (EC) and West Coast (WC). From this diagram it is clear that EUR has a vast amount of unique diversity (and overall diversity); that the WC is a large subset of the EC diversity; and that there is no direct connection between the WC and EUR given that there are no haplotypes just shared between the two regions.

Genetic analyses of the more recently-collected West Coast populations demonstrated reduced haplotype diversity compared to the East Coast, but both populations were dwarfed by European haplotype diversity (Fig. 4; Appendix S1). Among all regions, we observed a total of 131 haplotypes, but most were European, with only 25 haplotypes observed in North America (Fig. 4), 15 of which were observed in the West Coast.

Of these 15 West Coast haplotypes, 13 were shared with the East Coast and 10 with Europe (Appendix S2). Excluding the two unshared Anaheim haplotypes, all West Coast haplotypes were shared with the East Coast, while none were shared solely with Europe (Fig. 4). AMOVA analyses indicated no regional differentiation between the East and West Coasts (FCT = -0.00039; p = 0.41) as opposed to marginal differentiation between Europe and the West Coast (FCT = 0.02285; p = 0.09), indicating a closer connection between the East and West Coasts than between Europe and the West Coast. When pooling West Coast + East Coast haplotypes, 13 of 25 (52%) haplotypes are from the West Coast (Fig. 4), while among the Europe + East Coast haplotype pool, only 25 of 129 (19.4%) haplotypes are from the East Coast (Appendix S2).

**Figure 5:**
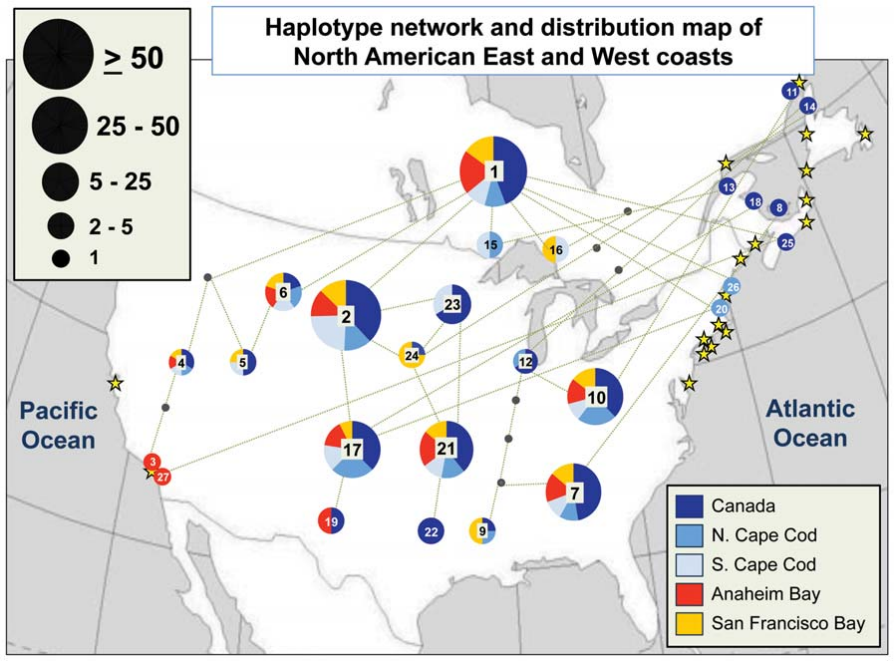
North American Haplotype Distribution Network Map. Yellow stars indicate sampling locations. Each haplotype is represented by a pie chart sized in proportion to its frequency in North American populations (out of a total of 347 individuals analyzed). Numbers on pie charts correspond to haplotype identity as listed in Appendix S1. Colors on the pie charts indicate the geographic distribution (for East Coast subregions and West Coast populations; see legend) of individuals with each haplotype. Lines between pie charts represent connections between haplotypes (based on a TCS 1.21 haplotype network), with the number of line segments indicating the number of sequence changes required to obtain a given haplotype from neighboring haplotypes (dots are intermediate nodes). Pie charts for haplotypes unique to a specific site are shown at that site's location.

Our haplotype network and distribution map (Fig. 5) illustrates the many connections between the East and West Coasts, especially for frequently occurring haplotypes. *L. littorea '*s genetic structure appears quite homogeneous, especially on the East Coast, where haplotypes were shared among individuals from throughout the eastern range. This homogeneity made it challenging to determine potential source populations from the East Coast for the introduced West Coast populations even when explored across larger potential source areas (Canada, US North of Cape Cod, and South of Cape Cod). Relatively high genetic diversity is also clearly present on the West Coast (Fig. 5).

**Figure 6:**
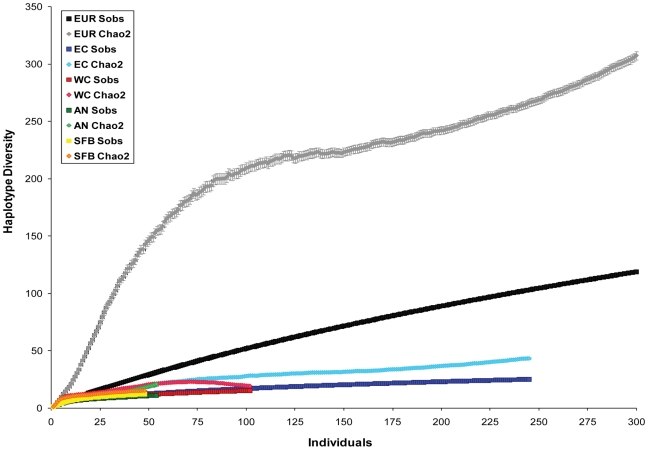
Rarefaction curves for Europe, East Coast North America, and West Coast North America. S_obs_ refers to the accumulation of observed haplotypes for the total number of individuals sampled in the European region (EUR S_obs_), East Coast (EC S_obs_), West Coast (WC S_obs_), Anaheim (AN S_obs_), and San Francisco Bay (SFB S_obs_). Chao2 predicts how many haplotypes were missing in the sampling of a particular region, including Europe (EUR Chao2), the East Coast (EC Chao2), the West Coast (WC Chao2), Anaheim (AN Chao2), and San Francisco Bay (SFB Chao2).

On the whole, the Europe to East Coast comparison represents a significant loss of haplotype diversity (χ^2^ = 70.23, df = 1, p<0.0001), while the East Coast to West Coast comparison does not (χ^2^ = 2.50, df = 1, p = 0.11). Our rarefaction analyses indicate that all three regions are undersampled, but to different degrees (Fig. 6). The West Coast estimator curve (WC Chao2) approaches an asymptote with the West Coast accumulation curve (WC Sobs), suggesting that few new haplotypes would be detected with additional sampling. The East Coast estimator curve (EC Chao2) appears at the beginning stages of asymptoting, while the European rarefaction curves indicate many more haplotypes would likely be found with further sampling. The similarity between the West Coast and East Coast curves contrasts with the marked separation between the East Coast and European rarefaction curves (Fig. 6).

#### East Coast source locations for the introduced West Coast populations

**Figure 7:**
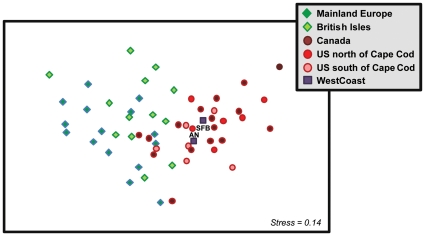
Multidimensional scaling plot of pairwise F_ST_ values for *Littorina littorea* in European, East Coast and West Coast populations. Europe is divided into two groups, Mainland Europe (dark green diamonds) and British Isles (light green diamonds). The East Coast is divided into Canadian (dark red circles), US north of Cape Cod (medium red circles), and US south of Cape Cod (light red circles) groupings. The two West Coast populations are represented by purple squares. These data show considerable spread among *Littorina littorea* populations and a large degree of mixing among all groups. West Coast populations are embedded among East Coast populations.

Determining precise source locations within the East Coast for the West Coast populations was challenging due to *Littorina littorea* 's spatially homogeneous genetic structure on the East Coast (Table 2, Fig. 5). An MDS plot based on pairwise F_ST_ data (Fig. 7) clearly demonstrates this lack of structure, with populations well spread out and a large degree of mixing among regions. The West Coast populations in particular are embedded among many different East Coast populations from Canada and the US, but spatially are not very close to any European populations. ANOSIM results corroborate these patterns: the East and West Coasts are very similar (R = 0.097, p = 0.308) while Europe and the West Coast show clear differences with little overlap (R = 0.452, p = 0.023).

**Table 2:** Sampling region and sites, sample size (N) and measures of genetic diversity within sampled populations.

Sampling Sites	Abbr.	N	a	b	h	π
EUROPE						
Moss, Norway	MO	10	4	4	0.533	0.0015
Tjarno, Sweden	TJ	9	4	5	0.482	0.0018
Varberg, Sweden	VA	4	3	6	0.833	0.0048
Copehagen, Denmark	CO	8	4	4	0.643	0.0021
Nyborg, Denmark	NY	10	5	7	0.667	0.0022
Ubdyhoj, Denmark	UB	8	5	7	0.857	0.0032
Esbjerg, Denmark	ES	9	9	11	1.000	0.0044
Ostende, Belgium	OS	10	8	11	0.933	0.0038
Scheldt Estuary, The Netherlands	SC	10	8	11	0.933	0.0040
Mindin, France	MI	10	7	10	0.867	0.0036
Roscoff, France	RO	16	12	18	0.958	0.0054
Trouville, France	TR	9	6	12	0.833	0.0047
Arcachon Bay, France	BD	10	7	9	0.911	0.0043
Vigo, Galicia, Spain	GA	11	10	13	0.982	0.0050
St Andrews, Scotland	ST	6	4	9	0.867	0.0052
Oban, Scotland	OB	14	10	22	0.890	0.0056
Peterhead, Scotlan	PH	16	11	18	0.875	0.0047
Greenock, Scotland	GR	22	16	24	0.948	0.0055
Fort William, Scotland	FtW	17	10	14	0.875	0.0047
Ullapool, Scotland	ULL	17	12	19	0.941	0.0056
Cromarty, Scotland	CR	12	12	14	0.833	0.0066
Cardigan Bay, Wales	CA	3	3	5	1.000	0.0053
Plymouth, England	PK	9	8	11	0.972	0.0047
Robin Hood Bay, England	RH	3	3	7	1.000	0.0075
Sligo, Ireland	SL	8	8	14	1.000	0.0069
Galway, Ireland	GW	10	5	4	0.667	0.0012
Limerick, Ireland	LI	10	5	13	0.844	0.0065
Dublin, Ireland	DU	10	9	10	0.978	0.0045
Cork, Ireland	CK	6	6	11	1.000	0.0065
**EAST COAST**						
Red Bay, Labrador	RB	12	8	15	0.924	0.0066
Blanc Sablon, Quebec	BS	9	4	14	0.917	0.0077
Point Mitis, Quebec	PM	8	5	7	0.857	0.0039
Isles de Mignan, Quebec	ID	4	3	3	0.833	0.0027
Flower's Cove, Newfoundland	FL	7	6	11	0.952	0.0073
Norris Point, Bonne Bay, Newfoundland	BO	10	6	11	0.844	0.0077
Searston, Newfoundland	SE	4	4	11	1.000	0.0099
Portugal Bay, Newfoundland	PB	6	5	10	0.933	0.0063
Saint Peter's Bay, Prince Edward Island, Canada	SP	9	6	12	0.889	0.0066
Bay du Vin, New Brunswick, Canada	NB	5	5	11	1.000	0.0074
Saint John, New Brunswick	SJ	9	4	4	0.778	0.0022
Pictou, Nova Scotia	PI	36	9	15	0.870	0.0064
Truro, Nova Scotia	TR	6	3	7	0.733	0.0058
North Sydney, Nova Scotia	NS	4	4	10	1.000	0.0093
Mulgrave, Nova Scotia	MU	6	5	7	0.933	0.0037
Halifax, Nova Scotia	HF	9	5	8	0.806	0.0034
Acadia, ME	AC	8	4	9	0.821	0.0070
Eastport, ME	EA	9	7	12	0.944	0.0067
Wells, ME	WE	9	6	12	0.889	0.0062
York, ME	YK	10	6	9	0.889	0.0054
Fort Stark, Newcastle, NH	FS	8	6	12	0.929	0.0065
Plymouth, MA	PL	7	11	11	1.000	0.0063
Monument Beach, Buzzards Bay, MA	BU	8	6	9	0.929	0.0048
Sengakontacket Pond, Martha's Vineyard, MA	SP	9	4	7	0.806	0.0040
Stonington, CT	ST	6	6	12	1.000	0.0074
Crane's Neck, Long Island, NY	CN	10	4	9	0.733	0.0034
Montauk, NY	MN	10	7	11	0.911	0.0064
Cape May, NJ	CM	6	4	9	0.800	0.0048
**WEST COAST**						
San Francisco Bay, CA	SFB	48	12	15	0.891	0.0060
Anaheim Bay, CA	AN	54	11	18	0.867	0.0060

*a*  =  number of haplotypes, *b*  =  number of polymorphic sites in the samples, *h*  =  haplotype diversity, π  =  nucleotide diversity.

## Discussion

Our demographic, genetic, and historical evidence suggest that the largest populations of *Littorina littorea* recently found on the North American West Coast are comprised of direct transplants of adults rather than established, self-sustaining populations. Demographic data indicate that these populations were most likely introduced via the live seafood trade (see discussion below), while our genetic analyses suggest the North American East Coast as the most likely source region (Fig. 7). Below, we discuss the demographic and genetic “fingerprint” of the West Coast *L. littorea* populations and its implications for understanding how, when, where, and why this failed invasion occurred. We then consider the larger implications of these West Coast populations for our understanding of invasion processes and vectors and their effect on the success or failure of new introductions.

### What stage of invasion?

The demographic data we collected show no evidence of recent successful recruitment of *Littorina littorea* in the three larger West Coast populations, although many snails were in reproductive condition, suggesting that they represent a very recent introduction without establishment (Fig. 3). If the observed populations were successfully reproducing and recruiting, a much broader range of size classes, including juveniles, should have been observed. For example, surveys in San Francisco Bay for *L. saxatilis,* an established congener also introduced from the East Coast, regularly turned up numerous specimens of all sizes of this smaller, morphologically distinct snail (Miller et al., unpublished). It appears likely that all sampled West Coast *L. littorea* individuals were actually East Coast snails that were transported to the West Coast, released, and subsequently removed during our collections. Because *L. littorea* can live at least 3 to 4 years [35], it is possible that some individuals had been in the system a few years prior to collection.

### The absence of a clear genetic bottleneck

Genetic bottlenecks, commonly observed when comparing recent introductions to source populations [36], were present in East Coast populations when compared to Europe (Appendix S2), but surprisingly, there was little evidence of bottlenecks in the West Coast populations compared to their putative East Coast source region (Figs. 4, 5). This result could be partly due to the apparent lack of self-recruitment in West Coast *L. littorea* populations revealed in our demographic analyses (Fig. 3), which obviates the possibility of post-establishment drift, selection, and other processes that often affect the genetic makeup of introduced populations [10], [12]. High levels of propagule pressure could also lessen or eliminate genetic bottlenecks, as discussed below.

Interestingly, our results contrast sharply with genetic diversity patterns of the European green crab, *Carcinus maenas*, which has a similar invasion history to *L. littorea* in North America [37], [38]. Both species appeared on the East Coast in the 1800s, possibly as accidental introductions from Europe in rock ballast [11], [39], and both display clear genetic bottlenecks on the East Coast compared to their native European ranges. However, in contrast to *L. littorea*, *C. maenas* has established self-sustaining populations on the West Coast and exhibits reduced genetic diversity there compared to the East Coast [40].

### Diversity and invasion history

Without reproduction, any genetic differences between the recipient population and putative source populations must have been imposed by the vector(s) transporting the species to the recipient area or caused by differential survival following arrival. Any vector transporting non-native species essentially takes a sample of the available source regions' genetic diversity and transports it to the recipient region. Pre-establishment factors that can influence an invading species' population genetics include the number of introduction events, the number of invaders per event, and the effective size of the founding population(s) [10].

Due to the lack of a clear genetic bottleneck, West Coast *Littorina littorea* appeared to display signatures of a ‘genetic paradox,’ a term labeling cases where typical founder effects can be countered by additional diversity brought in through large founding or multiple introduction events [10], [41]. Although West Coast *L. littorea* populations do not appear established, the relatively high genetic diversity we observed could still have resulted from multiple inoculation events transporting a variety of haplotypes, possibly from different regions.

High genetic diversity at small spatial scales within the source region could also result in the transplantation of a substantial amount of diversity even from one or a few introduction events. East Coast haplotype diversity was relatively high (Table 2) across populations (though still significantly lower than Europe), suggesting that East Coast populations are comprised of many different haplotypes represented across the region (Figs. 5, 7; Appendix S1). It is therefore challenging to determine whether the West Coast populations resulted from a few or multiple inoculation events, though the latter seems the most likely scenario given that the probable vector is still operating (see below) and that the introduced populations of the species occur in multiple, widely spaced populations (i.e., San Francisco Bay and Anaheim Bay, which are hundreds of kilometers apart).

### How did *Littorina littorea* arrive on the West Coast?

While several vectors may have transported *Littorina littorea* to the West Coast, we suggest that the live seafood trade is likely responsible for the populations described here. Live adult snails are collected on the East Coast, particularly in New England, shipped to the West Coast, and sold in seafood markets (J. Carlton, pers. comm.). While snails may have been collected in Europe and shipped to the West Coast, this seems economically infeasible, and our genetic analyses show that West Coast populations were more closely aligned with the East Coast than Europe (Fig. 7). Because larger snails are more marketable, whether for food or bait, this vector necessarily excludes juvenile snails and thus seems a good candidate for producing the observed size-frequency distributions of the three large populations (Fig. 3). Informal surveys of Asian markets around the San Francisco Bay region revealed numerous stores selling adult *L. littorea*, often in 1-lb bags marked as “live sea snails” (A. Chang, pers. obs.) All three populations reported here were discovered in popular, easily accessible locations within 10 km of numerous seafood markets and bait shops.

There are several ways in which *L. littorea* might have been taken from the market and released into the wild, including disposal of unwanted snails originally intended for consumption or use as bait, or an intentional attempt to start a local fishery. Another possibility is *fangsheng*, also known as “releasing life” or “prayer-animal-release,” in which live animals are purchased from a market and released in accordance with a religious belief that one can accrue merits by releasing captive animals into the wild [42], [43]. In accordance with the belief in saving animal lives that drives this practice, the released animals can include snails and insects as well as “charismatic” animals such as birds, turtles, and fish [43], [44].

Consideration of demographic, genetic, and historical data rules out other potential vectors for transporting *Littorina littorea* to the West Coast. Although Eastern oyster (*Crassostrea virginica*) transplants were historically a major vector delivering numerous estuarine and marine species from the East Coast to the West Coast, this vector has been largely inactive since the early 1900s [45]. Given the level of sampling effort over the years, combined with the observed spatial extent and demographic data (size structure), it is improbable that established *L. littorea* populations would remain undiscovered for so long.

Ballast water is an unlikely vector for transporting *Littorina littorea* to the West Coast from either the East Coast or Europe because vessels arriving to the US West Coast primarily come from Asia (where *L. littorea* is absent), rather than the Atlantic [46]. One or a few individuals could have been introduced via ballast water from the East Coast or Europe and grown to adult size before being discovered. However, it is highly improbable that significant numbers of *L. littorea* larvae would be taken up by ship ballasting operations in the source region, transported to San Francisco and Anaheim Bays, survive, metamorphose, and settle only in the small (50–100 m) stretches of shoreline where we observed them.

Another improbable vector for the three large *Littorina littorea* populations in California is transport in algal packaging materials. The Atlantic alga *Ascophyllum nodosum* is frequently used as packaging material for baitworm and lobster shipments from New England to various domestic and international locations [47]. *Ascophyllum* itself has occasionally been introduced to California, and several species frequently found in baitworm shipments, including *Carcinus maenas* and *L. saxatilis*, may have been introduced to the San Francisco Bay region this way [25], [37], [47]. However, *L. littorea* densities in algal packaging materials are relatively low and consist of a range of sizes corresponding to those found in the New England intertidal habitats where *Ascophyllum* is collected, making this an unlikely vector for the large, concentrated populations of uniformly adult size described here (A. Chang, A. Blakeslee, W. Miller, G.Ruiz, pers. obs.). Nevertheless, because this vector is still active, small numbers of individuals may be introduced this way.

### Why has *Littorina littorea* failed to establish self-sustaining populations on the West Coast?

*Littorina littorea* is considered a failed invader of the West Coast despite a long history of successful transport from the Atlantic Coast and seemingly favorable climatic matching between the Atlantic and the West Coast [45]. We argue that the snail's most significant barriers to successful establishment on the West Coast are related to reproduction and recruitment. *L. littorea* likely had many opportunities to reproduce in the populations described here, given that numerous individuals in reproductive condition were found congregated together and could have been present for months to years prior to removal. The presence of vast rocky substrate and less extreme temperature regimes than those occurring in the snail's north Atlantic habitats suggest that physical environmental factors should not prevent its establishment or subsequent spread across much of the Pacific Coast of North America.

However, Wells (1965) [48] suggested that high water temperatures might limit *L. littorea* 's spread in the Atlantic, noting that its southern latitudinal limit on the East Coast and European shores occurred where the summer monthly mean water temperature reaches at least 21°C. Although the highest monthly mean water temperatures outside embayments on the temperate Pacific Coast are typically much lower than this, regional climatic variation produces monthly mean water temperatures in excess of 21°C in some parts of both San Francisco and Anaheim Bays. The warmest monthly mean temperature at Dumbarton Pier is 21.8°C in August, averaged across 2005–2009 [49], and approximately 24°C in July and August in Anaheim Bay (A. Chang, unpublished data; [50]). Significant temperature variation occurs throughout both bays, so even if high temperatures retard *L. littorea* 's establishment in some areas, introductions to nearby locations may face no such barrier. For example, the maximum monthly mean temperature at Fort Point (which is next to a public fishing pier) at the mouth of San Francisco Bay was 16.8°C during 2007–2009 [51].

Broadcast spawners such as *L. littorea* may also be more likely to experience Allee effects than brooding and capsule-laying snails when introduced to a new location [8], [22], [45]. This explanation has been invoked for the absence of *L. littorea* in certain European locations where its congener *L. saxatilis* (which has crawl-away larvae) is established [22]. Johannesson (1988) [22] suggested that although both *L. saxatilis* and *L. littorea* could disperse equally well via crawling, rafting, and wave transport, *L. saxatilis* has an advantage in retaining its place in new locations because its crawl-away larvae allow swift colonization of new areas in some abundance, whereas *L. littorea* would need to rely on repeated transport of its planktonic larvae to new locations in order to establish a foothold. Similarly, in West Coast populations of *L. littorea*, larvae released into the water column may end up settling in locations far from congregated populations of the snail—thus impeding the creation of self-sustaining, established populations in this region. However, it is also possible that such Allee effects may be counteracted by greater propagule pressure due to continued operation of vectors including the live seafood trade that actively select and transport large numbers of adult *L. littorea* to the Pacific Coast, especially if introduced to a region in sufficient quantities in conditions favorable to reproduction and larval retention.

### Conclusions

We have taken advantage of a rare opportunity to document the genetic and demographic signatures of several newly discovered populations of a non-native species at a very early stage of invasion, specifically prior to successful establishment of self-sustaining populations. We used these signatures to infer the likely vector and source region of West Coast populations of *Littorina littorea*, revealing several important facets of the invasion process and the probable signature of live trade vectors on the resulting introduced populations. First, the transport of large individuals rather than larvae or juveniles, combined with an apparent absence of recruitment, probably resulted in populations with an unusual size structure consisting only of adults. A consequence of this preferential transport of adults could be an enhanced risk of introducing associated parasites and diseases [52] since parasite loads of gastropods, and specifically *L. littorea*, increase with age (which is correlated with size) [53]. Second, the genetic diversity of West Coast *L. littorea* described here was not significantly reduced compared to its putative East Coast source. This is likely due, at least in part, to the homogeneous genetic structure across East Coast populations, as well as the lack of evolutionary processes in the non-established West Coast populations that might otherwise act to lower diversity. Third, local climatic variation can significantly impact the likelihood of invasion success and may have been responsible for the failure of West Coast *L. littorea* invasions. Since the numerous vectors delivering *L. littorea* to West Coast shorelines operate over a much larger region than the local areas with apparently unfavorably high temperatures where these populations were found, continued introductions to other areas may well result in successful invasion. Subsequent introductions may also serve to reduce the impact that Allee effects may be having on these West Coast populations. Finally, live seafood trade vectors deliver large numbers of adults of many species in good condition, providing unique opportunities for invasion. Control measures are crucial to reducing the risks posed by these vectors, especially since this trade is predicted to increase with growing worldwide demand for aquaculture [54]. The ongoing operation of this and other vectors is highlighted by recent discoveries – in greater numbers than ever found before – of the northern Atlantic periwinkle *Littorina littorea* in the already highly invaded San Francisco and Anaheim Bays.

## Supporting Information

Appendix S1 **Total number of haplotypes (HAP) found in the East Coast (EC) and West Coast (WC) of North America and all European haplotypes.** Total numbers (N) for each site are at the bottom and haplotype frequencies (Overall, WC, EC, and EUR) are in the right most columns. Location refers to regions in which haplotypes were found. Site abbreviations correspond to those in Table 2.
(XLS)

Appendix S2 Total number of haplotypes (HAP) found in the East Coast (EC) and West Coast (WC) of North America and those haplotypes that are also shared with Europe (EU*). Total numbers (N) for each site are at the bottom and haplotype frequencies (Overall, EU*, EC, WC, and EC & WC) are in the right most columns. *Only European haplotypes that are shared with North American haplotypes are included here; there are an additional 104 haplotypes from 146 individuals found just in Europe (see Appendix S1). Location refers to regions in which haplotypes were found. Site abbreviations correspond to those in Table 2.
(XLS)
